# Feasibility of a single mediastinal drain through the abdominal wall after esophagectomy

**DOI:** 10.1097/MD.0000000000013234

**Published:** 2018-11-16

**Authors:** Yan Zheng, Yin Li, Xianben Liu, Ruixiang Zhang, Zongfei Wang, Haibo Sun

**Affiliations:** a Department of Thoracic Surgery, The Affiliated Cancer Hospital of Zhengzhou University, Henan Cancer Hospital, Zhengzhou, Henan; b Department of Thoracic Surgery, National Cancer Center/National Clinical Research Center for Cancer/Cancer Hospital, Chinese Academy of Medical Sciences and Peking Union Medical College, Beijing, China.

**Keywords:** fast-track surgery program, mediastinal drainage tube, minimally invasive esophagectomy

## Abstract

This study evaluated the safety and effectiveness of a single mediastinal drainage tube in the thoracic and abdominal cavity after minimally invasive esophagectomy (MIE). This study was undertaken to determine if the procedure could be included in a fast-track surgery program for resectable esophageal carcinoma (EC).

From June 17 to November 30, 2015, clinical data for 78 eligible patients who had undergone a fast-track surgery program and MIE were retrospectively analyzed. Twenty-eight patients had a chest tube and mediastinal drainage tube. Thirty-four patients had only a mediastinal drainage tube through the intercostal space. The remaining 30 patients had a single mediastinal drainage tube in the thoracic and abdominal cavity through the abdominal wall. The complication rates and pain scores for each of the groups were compared. The statistical calculations were performed using SPSS 17.0 for Windows (SPSS Inc., Chicago, IL). The quantitative data among the groups were compared using 1-way analysis of variance (ANOVA). The Chi-square, Mann–Whitney *U* and Fisher exact tests were used for qualitative data analysis.

There were no significant differences in the anastomotic leak rates, postoperative days and total complication rates (*P* = .861). The lowest visual analog scale (VAS) scores of the drainage tubes were observed in the group with a single mediastinal drain through the abdominal wall (*P* <.001).

The results of this study suggested that a single mediastinal drainage tube in the thoracic and abdominal cavity after MIE may be safe and efficient. This clinical practice is a part of our fast-track surgery program.

## 1 Introduction

Esophageal carcinoma (EC) is the eighth most common cancer and occurs commonly in less-developed regions of the world.^[1]^ In 2012, there were 456,000 new cases and 400,000 deaths due to esophageal squamous cell carcinoma (ESCC).^[1–3]^ Esophagectomy is associated with high complication rates, a long postoperative stay and slow recovery of baseline activity levels.^[4]^ Focusing on the quality of life (QOL), financial savings and fast recovery, we have previously evaluated a “no tube, no-fasting” fast-track surgery program for resectable EC.^[5]^ The key points in this program were as follows:

1. All esophageal cancer patients receive minimally invasive esophagectomy (MIE).
2. The patients had 1 mediastinal drainage tube after esophagectomy without a chest drainage tube, jejunum nutrition tube or nasogastric tube. It was referred to as “no tube”.
3. The “no fasting” means the patients start oral feeding at will on postoperative day 1 (POD1).^[5,6]^

The management of chest drainage is a pivotal part of the “no tube, no fasting” fast-track surgery program. This study only focused on this topic and evaluated the safety and effectiveness of a single mediastinal drainage tube in the thoracic and abdominal cavity without a chest tube.

The chest tube is known to be 1 of the most important factors influencing hospital stay, postoperative pain, and costs.^[7,8]^ Many studies have focused on evaluating different management approaches to chest tubes, such as suction or no-suction, single or double chest tube and the removal of chest tubes.^[9]^ However, because of the high morbidity rate of esophagectomy, few studies have focused on the fast-track management of chest tubes. As one of the most important parts of the “no tube, no fasting” fast-track program,^[5]^ the present study attempted to reduce the number of chest drainages and find the best location for the chest drainage.

We hypothesized that a single mediastinal drainage tube through the abdominal wall would reduce pain and would not increase the complication rates of esophagectomy. Against this background, this retrospective study assessed the pain and postoperative complication rates of 3 different chest drainage management approaches.

## 2 Materials and methods

Patient data were collected at the thoracic surgery department of the Henan Cancer Hospital. From June 17 to December 31, 2015, clinical data for 78 eligible patients were retrospectively collected and analyzed. The study was approved by the Institutional Review Board and the Ethics Review Committee of Henan Cancer Hospital. All patients underwent McKeown MIE with 2-field or 3-field lymph node dissection. All operations were performed by experienced surgeons. During the operation, 4 ports were made in the chest wall, and 5 were made in the abdominal wall. The camera port was located in the seventh intercostal space (ICS) between the anterior axillary line and the mid-axillary line. A 5-mm instrument port was placed at the ninth ICS in the posterior axillary line. The mediastinal tube was a gastric tube (type II-4.67 mm Fr 14) with handmade double holes every 3 cm from the beginning to 25 cm. The chest tube was removed on POD1. The mediastinal tube was designed to be removed on the morning of POD5 if there was no hydropneumothorax in the chest CT scan on POD4 and the drainage volume was less than 300 mL. The flurbiprofen acetate injection (50 mg tid, POD1–3) was intravenously administered. If the pain scores were higher than 3, another regime, such as tramadol, was used. There was no intercostal nerve block. All of the patients started oral feeding on POD1 and discharged home on POD5–7.

**Figure 1:**
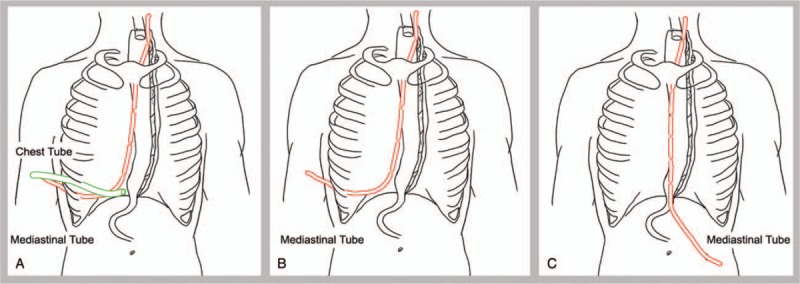
Graphs of the 3 different chest drainages. Picture A shows the chest drainage management of Group A. There were 2 chest drainage tubes. The green tube was a 1-cm chest tube at the seventh ICS between the anterior axillary line and the midaxillary line. The red tube with double holes every 3 cm was a 5-mm mediastinal drainage tube in the ninth ICS along the posterior axillary line. Picture B represents the chest drainage management of Group B. Group B had only 1 red mediastinal drainage tube in the same location and of the same size as Group A. Picture C shows the management of chest drainage of Group C. The mediastinal drainage tube was inserted through a 5-mm instrument hole that was located in the left lower abdominal wall. The tube went through the esophageal hiatus from the abdominal cavity to the thoracic cavity. We made double holes in the mediastinal tube every 3 cm from the beginning of the tube to the tube close to the esophageal hiatus. The length of the mediastinal tube from the beginning of the tube to the esophageal hiatus was approximately 25 cm. ICS =  intercostal space.

From June 17 to August 11, 28 patients who had a thoracic cavity drainage tube and a mediastinal drainage tube were recruited. A 1-cm chest tube was placed through the camera port. A 5-mm mediastinal drainage tube was inserted through the instrument port. Patients who received this procedure were classified as Group A. From August 19 to October 9, 34 patients who had a mediastinal drainage tube through an instrument port were recruited into the study as Group B. Compared with Group A, we did not put in a chest tube and retained the mediastinal tube. From October 9 to December 31, there were 30 patients who had only a single mediastinal drainage tube in the thoracic and abdominal cavities. The tube was inserted through a 5-mm instrument hole that was located in the left lower abdominal wall. Patients who received this procedure were classified as Group C. Graphs of the 3 different chest drainage protocols are shown in Figure 1.

The following surgical and demographic data were collected: age, gender, tumor location, neoadjuvant therapy, operation time, lymphadenectomy, number of chest tubes, duration of chest tubes, histology, and pathological stage. These data were compared with baseline information. Postoperative cardiac complications, respiratory complications, gastrointestinal complications, recurrent need for intensive care unit (ICU) treatment and VAS scores for pain were used as outcome variables. The VAS of drainage-associated pain scores was collected by the research nurse every day at 11 am before the drainage tubes were removed. If patients averaged 5 to 6 days of mediastinal drainage, the scores were averaged over that time frame. Data from the 3 groups were compared. The statistical calculations were done using SPSS 17.0 for Windows (SPSS Inc., Chicago, IL). The quantitative data among the groups were compared using 1-way analysis of variance (ANOVA). The Chi-square, Mann–Whitney *U* and Fisher exact tests were used for qualitative data. The *P* value was considered to be statistically significant at .05.

## 3 Results

**Figure 2:**
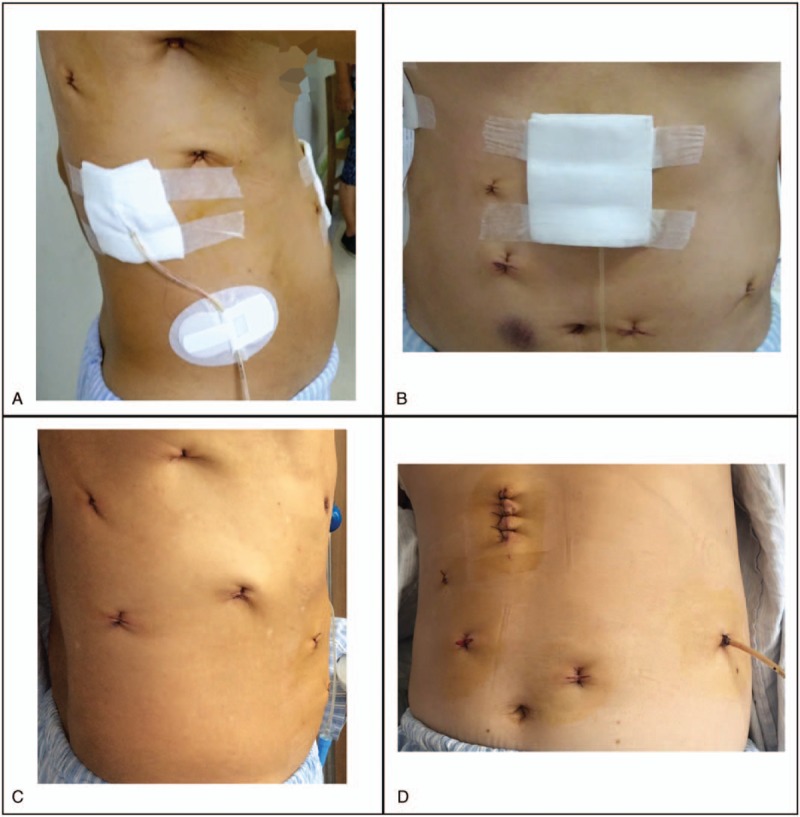
Pictures of patients and the location of mediastinal tubes from Group B and Group C on POD1 (A) and (B), pictures of patients in Group B; (C) and (D), pictures of patients in Group C. (A) and (C) show the incisions in the chest wall. (B) and (D) show the incisions in the abdominal wall. (A) and (D) show the location of the mediastinal tubes in each group. POD1 = postoperative day 1.

There were no statistically significant differences in the surgical data among the 3 groups. The details are shown in Table 1. The outcome variables (postoperative cardiac complications, respiratory complications, gastrointestinal complications, and recurrent need for ICU treatment) were comparable. The drainage-associated VAS pain scores were significantly different among the 3 groups. The mean scores of Groups A, B, and C were 2.68 ± 0.61, 1.76 ± 0.55, and 1.27 ± 0.58, respectively (*P* <.001). The outcomes are shown in Table 2. Figure 2 shows the location of mediastinal drainage tubes in Group B and Group C on POD1.

**Table 1:**
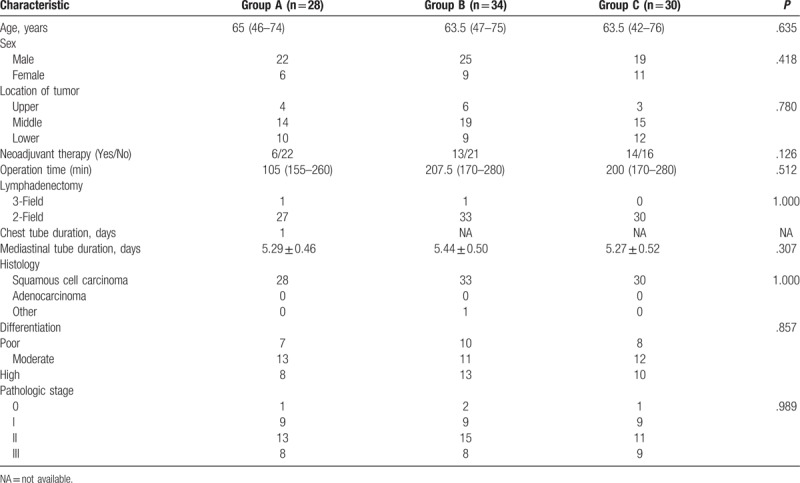
Baseline comparison of treatment groups.

**Table 2:**
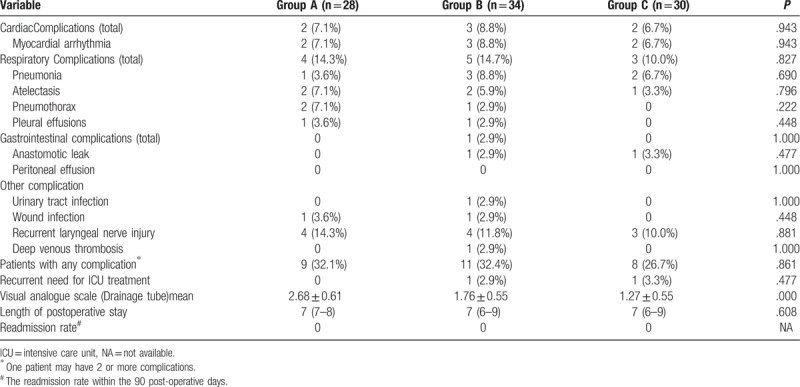
Perioperative outcome among the 3 groups.

## 4 Discussion

Our retrospective data have shown that a single mediastinal drainage tube in the thoracic and abdominal cavity through the abdominal wall after MIE did not increase postoperative complications. Compared with the other 2 chest drainage protocols, it could significantly reduce drainage-associated pain and should be a standard chest drainage management protocol in our fast-track surgery program.^[5]^

All of the patients in the first ward of our department had the same clinical pathway. In this study, only 2 patients in Group A had average pain scores of 4 and were administered tramadol. The pain control was comparable in the 3 groups. There were concerns about the possibility that the mediastinal tube was causing pneumothoraxes; therefore, on the day of the operation, the mediastinal tubes of patients in Groups B and C were linked to a water-sealed bottle. On the morning of POD1, the mediastinal tube was linked to a negative pressure-absorbing ball rather than the water-sealed bottle. Pneumothorax was not observed in any of the patients. However, more patients in Group B had hydrothorax in the left thoracic cavity. Two of them received thoracentesis. Patients in Group B had only a single mediastinal tube through the chest wall. The hydrothorax in the left costophrenic angle was not easy to drain. However, moving the tube through the esophageal hiatus, like for Group C, ensured a drainage balance on both sides of the thoracic cavity.

Chest drainage management has become a hot topic for thoracic surgeons who perform fast-track surgery programs. We tried to find the best chest drainage protocol to include in our fast-track surgery program. Many studies have tried to evaluate the best strategy for chest drainage management after lung surgery. These studies have focused on air leakage duration.^[10]^ However, after esophagectomy, the incidence of air leaks is much lower. The main problem is the drainage of the hydrothorax.^[11]^ For the mediastinal tube, we made double holes every 3 centimeters from the beginning to 25 cm to avoid blockage and to ensure drainage from the top aside the anastomosis and the lowest part of the thoracic cavity.

A numeric pain scale was adopted to assess the degree of pain before the removal of the chest tube. In clinical practice, the numeric pain scale is recognized as a preferred pain measurement tool.^[12]^ A 10-degree picture scale, such as the visual analog scale (VAS), is an easy tool for assessing pain.^[13]^ However, the numeric pain scale does not provide data on the quality of pain. Chest tube pain may also be influenced by the thoracotomy incision. Thus, the present study included only the MIE surgery. In a previous study, it was reported that postoperative pain may be caused by compression of the intercostal nerve.^[14]^ Another study suggested that the chest tube in the ICS insertion may lead to intercostal nerve impairment.^[15]^ In the present study, the chest drainage protocol of Group C avoided continued squeezing of the intercostal nerves. This finding may be the reason why patients in Group C had the lowest pain scores.

There were several possible limitations that the present study may have. First, as this study was retrospective, there could have been a selection bias. Second, the sample size was small. However, at present, the protocols for Groups B and C are used daily in our department. In particular, the protocol used for patients in Group C has been more widely adopted.

## 5 Conclusions

This study suggested that a single mediastinal drainage tube in the thoracic and abdominal cavity after MIE may be safe and may reduce chest pain. However, further data are needed to evaluate the economic implications, the reduction in the nurses’ workload, and the QOL index for patients.

## Author contributions

**Conceptualization:** Yin Li.

**Data curation:** Yin Li, Xianben Liu, Ruixiang Zhang.

**Formal analysis:** Xianben Liu.

**Funding acquisition:** Yan Zheng.

**Investigation:** Xianben Liu.

**Methodology:** Yan Zheng.

**Resources:** Yin Li, Xianben Liu, Zongfei Wang.

**Software:** Ruixiang Zhang.

**Supervision:** Xianben Liu.

**Validation:** Haibo Sun.

**Writing – original draft:** Yan Zheng.

**Writing – review & editing:** Yin Li, Xianben Liu, Ruixiang Zhang, Zongfei Wang, Haibo Sun.
